# Specifications of standards in systems and synthetic biology: status, developments, and tools in 2024

**DOI:** 10.1515/jib-2024-0015

**Published:** 2024-07-22

**Authors:** Martin Golebiewski, Gary Bader, Padraig Gleeson, Thomas E. Gorochowski, Sarah M. Keating, Matthias König, Chris J. Myers, David P. Nickerson, Björn Sommer, Dagmar Waltemath, Falk Schreiber

**Affiliations:** 40092 Heidelberg Institute for Theoretical Studies (HITS), Heidelberg, Germany; University of Toronto, Toronto, Canada; Dept. of Neuroscience, Physiology and Pharmacology, University College London, London, UK; School of Biological Sciences, University of Bristol, Bristol, UK; Advanced Research Computing Centre, University College London, London, UK; 9373 Institute for Biology, Institute for Theoretical Biology, Humboldt-University Berlin, Berlin, Germany; Dept. of Electrical, Computer, and Energy Eng., University of Colorado Boulder, Boulder, USA; 428614 Auckland Bioengineering Institute, University of Auckland, Auckland, New Zealand; 4910 Royal College of Art, London, UK; Medical Informatics Laboratory, University Medicine Greifswald, Greifswald, Germany; Dept. of Computer and Information Science, 26567 University of Konstanz, Konstanz, Germany; Faculty of Information Technology, Monash University, Clayton, Australia

## 1 Introduction

The “COmputational Modeling in BIology NEtwork” (COMBINE) initiative aims to harmonise the development of diverse community standards for computational models in biology [1, 2]. It coordinates standard development to support the associated projects towards establishing a suite of compatible, interoperable and comprehensive standards that address the full spectrum of modeling in systems and synthetic biology.

**Figure 1:**
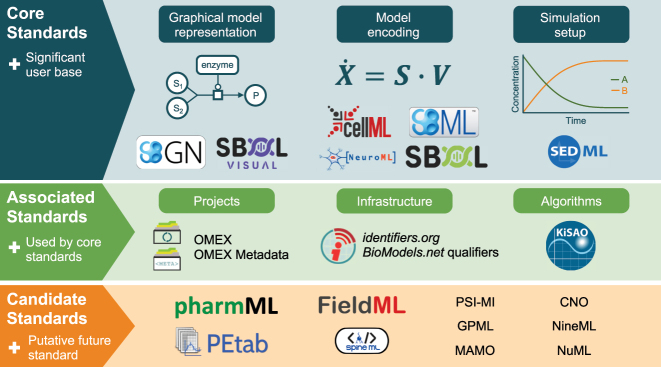
COMBINE standards and associated efforts (image taken from [3]).

Figure 1 provides a comprehensive view of the COMBINE standards along with related efforts. Special issues focusing on COMBINE standards have been released regularly since 2016, offering updates from 2015 through 2023 as documented in [3–9].

This editorial discusses the most recent updates to COMBINE standards, showcasing the advancements made over the past year. Specifically, it introduces a new specification: the Simulation Experiment Description Markup Language (SED-ML) Level 1 Version 5. Furthermore, this editorial briefly summarises the key points of the standards and links to software (tools) that are utilising these standards or are important to their implementation, including three new tools:

– SBMLToolkit.jl [10], a Julia package for importing SBML into the SciML ecosystem,
– MakeSBML [11]: A tool for converting between Antimony and SBML, and
– MetaLo [12], metabolic analysis of logical models extracted from molecular interaction maps.

## 2 Current versions of COMBINE standards

In this issue, similar to our past special issues, we will provide a concise summary of all COMBINE standards. For the most current specifications of COMBINE standards, please consult the sections that follow. Any new specifications or updates to existing ones are marked with **NEW**. To facilitate ease of navigation, the structure and core information remain consistent with previous special issues.

### 2.1 Core standards

A comprehensive overview about the COMBINE core standards, that can be interactively searched an browsed, can be found as COMBINE collection in the FAIRsharing platform (https://fairsharing.org/3495). The COMBINE core standards are also referenced by several standards recently published by the International Organization for Standardization (ISO). ISO 20691:2022 “Biotechnology – Requirements for data formatting and description in the life sciences” (https://www.iso.org/standard/68848.html) provides recommendations and requirements for the model formatting, as well as for the semantic description and annotation of data and models in the life sciences, and recommends the COMBINE core standards in its annex. ISO/TS 9491-1:2023 “Biotechnology – Recommendations and requirements for predictive computational models in personalised medicine research – Part 1: Guidelines for constructing, verifying and validating models” (https://www.iso.org/standard/83516.html) specifies requirements and recommendations for models used for research purposes in the field of personalised medicine and provides guidelines to apply the COMBINE core standards in that field.

#### 2.1.1 BioPAX (Biological PAthway eXchange)

BioPAX is a language designed for the integration, exchange, and analysis of biological pathway data. It utilises OWL for its expression. The current specification is:

**Table:** 

Standard	Specification	Reference
BioPAX [13]	BioPAX	[14]

Tools for BioPAX include Paxtools [15], PathVisio [16], and ChiBE [17].

#### 2.1.2 CellML

The CellML language is an XML-based markup language designed for the storage and exchange of computer-based mathematical models. The current specifications are:

**Table:** 

Standard	Specification	Reference
CellML [18]	CellML 2.0.1	[19]
	CellML Metadata Framework 2.0	[20]

The CellML Metadata Framework [20] is now deprecated in favour of the OMEX Metadata Specification [21]. Tools for CellML include libCellML (https://libcellml.org) and OpenCOR [22]. A tool overview can be found at https://cellml.org/tools.

#### 2.1.3 NeuroML

The Neural Open Markup Language (NeuroML) is a description language based on XML, offering a standardised data format for the definition and exchange of neuronal cell and network model descriptions. The current specification is:

**Table:** 

Standard	Specification	Reference
NeuroML [23, 24]	NeuroML version 2.3	[23]

Tools for NeuroML include jNeuroML [23], NetPyNE [25], and EDEN [26]. A tool overview can be found at https://docs.neuroml.org/Userdocs/Software/Software.

#### 2.1.4 SBGN (Systems Biology Graphical Notation)

The Systems Biology Graphical Notation (SBGN) provides a suite of standardised graphical languages designed for visually representing biological knowledge. It encompasses three distinct languages, Process Description, Entity Relationship, and Activity Flow. Furthermore, SBGN-ML, an XML-based file format, is utilised for detailing the geometry of SBGN maps. The current specifications are:

**Table:** 

Standard	Specification	Reference
SBGN [27]	SBGN Process Description Level 1 Version 2	[28]
	SBGN Entity Relationship Level 1 Version 2.0	[29]
	SBGN Activity Flow Level 1 Version 1.2	[30]
	SBGN Markup Language Version 0.3	[31]

Tools for SBGN include CySBGN [32], PathVisio (SBGN plugin) [16], and SBGN-ED [33]. A tool overview can be found at https://sbgn.github.io/, as well as in [34].

#### 2.1.5 SBML (Systems Biology Markup Language)

The Systems Biology Markup Language (SBML) [35, 36] is an XML-based format designed for computer interpretation of models of biological processes. While it is especially suited for models that describe processes, SBML’s application is not confined to these alone. The current specifications are:

**Table:** 

Standard	Specification	Reference
SBML [37]	SBML Level 3 Core, Version 2, Release 2	[35]
	SBML Level 3 Package: Distributions, Version 1, Release 1	[38]
	SBML Level 3 Package: Flux Balance Constraints	[39]
	Version 3, Release 1 SBML Level 3 Package: Groups, Version 1, Release 1	[40]
	SBML Level 3 Package: Hierarchical Model Composition, Version 1, Release 3	[41]
	SBML Level 3 Package: Layout, Version 1, Release 1	[42]
	SBML Level 3 Package: Multistate, Multicomponent and Multicompartment Species, Version 1, Release 2	[43]
	SBML Level 3 Package: Spatial Processes, Version 1, Release 1	[44]
	SBML Level 3 Package: Qualitative Models, Version 1, Release 1	[45]
	SBML Level 3 Package: Render, Version 1, Release 1, Release 1	[46]

Tools for SBML include COPASI [47], roadrunner [48, 49], CySBML [50] (https://sbml4humans.de) and sbmlutils [51]. A tool and model overview can be found at https://sbml.org/software/.

#### 2.1.6 SBOL (Synthetic Biology Open Language)

The Synthetic Biology Open Language (SBOL) is a language for detailing and sharing information about synthetic biological components, devices, and systems. SBOL Visual (SBOLv), a related standard, offers a uniform collection of symbols and guidelines for illustrating genetic circuits. The current specifications are:

**Table:** 

Standard	Specification	Reference
SBOL [52]	SBOL Version 3.1.0	[53]
	SBOL Visual Version 2.3	[54]
	SBOL Visual Version 3.0	[55]

Tools for SBOL and SBOL Visual include SynBioHub [56], SBOLCanvas [57], DNAplotlib [58], paraSBOLv [59] and VisBOL [60]. A tool overview can be found at https://sbolstandard.org, as well as in [61].

#### 2.1.7 SED-ML (Simulation Experiment Description Markup Language)

The Simulation Experiment Description Markup Language (SED-ML) is a format based on XML that is used for detailing simulation experiments. It enables the specification of which models to use, the experimental tasks to execute, and the results to generate. SED-ML supports models that are encoded in a variety of languages. The current specification is:

**NEW** The Simulation Experiment Description Markup Language (SED-ML): Language Specification for Level 1 Version 5 [62] enhances the capabilities for modelers to specify simulations within SED-ML through the Kinetic Simulation Algorithm Ontology (KiSAO). Although it was already feasible to specify a simulation with KiSAO in Version 4, the new version extends this capability, enabling users to also utilise the ontology for defining tasks, model modifications, ranges and outputs.

**Table:** 

Standard	Specification	Reference
SED-ML [63]	SED-ML Level 1 Version 5	[62]

Tools for SED-ML include many of the tools listed on BioSimulators (https://biosimulators.org/) and COPASI [47]. A tool overview can be found at https://sed-ml.org/showcase.html.

### 2.2 Associated standards

Associated standards provide an additional layer of semantics to COMBINE representation formats. The current specifications are:

**Table:** 

Standard	Specification	Reference
COMBINE Archive [64]	COMBINE Archive 1.0	[65]
OMEX Metadata	OMEX Metadata Version 1.2	[21]
BioModels.net qualifiers [66]	–	[67]
Identifiers.org URIs [68]	–	[69]
Systems Biology Ontology [70]	[External] Bioportal	[71]
Kinetic Simulation Algorithm Ontology [70]	[External] Bioportal	[72]

A COMBINE archive consolidates multiple documents and essential information required for a modelling and simulation project into a single file. This archive utilises the Open Modeling EXchange (OMEX) format for encoding. The COMBINE archive metadata offers a unified, community-endorsed method for annotating diverse standardised model and data formats contained within a COMBINE archive.

BioModels.net qualifiers represent standardised relationships (predicates) that define the connection between an object in a descriptive language and the external resource used for its annotation. MIRIAM Unique Resource Identifiers (URIs) enable the unique and unambiguous identification of an entity in a consistent and lasting way. The MIRIAM Registry offers a set of services and resources that assist in creating, understanding, and resolving MIRIAM URIs. Using Identifiers.org technology, MIRIAM URIs can be accessed in a versatile and reliable manner. These URIs are used by controlled annotation schemes in SBML, SED-ML, CellML, and BioPAX.

The Systems Biology Ontology (SBO) comprises a collection of controlled, relational vocabularies encompassing terms frequently used in Systems Biology, especially within the realm of computational modelling. Every component within an SBML (Systems Biology Markup Language) file may include an optional attribute named sboTerm, which should correspond to a specific term from the SBO. Furthermore, every symbol used in SBGN (Systems Biology Graphical Notation) is linked to an appropriate term from the SBO.

The Kinetic Simulation Algorithm Ontology (KiSAO) describes various algorithms along with their characteristics and the relationships between them through their specific features and parameters. It is utilised within the Simulation Experiment Description Markup Language (SED-ML), enabling simulation software to automatically select the optimal algorithm for a given simulation and unambiguously refer to it.

The OMEX Metadata Specification serves as a technical implementation of the community consensus among COMBINE standards, aimed at standardising the description of computational models and other resources through metadata, as outlined by [73].

### 2.3 Tools

To work with COMBINE standards, various tools and software are available, designed to support different aspects of modeling and simulation, as well as corresponding data/model integration and data management (see the previous Section). This special issue introduces a few new tools:

– SBMLToolkit.jl [10] is a tool designed to bridge the gap between systems biology and the advanced computational capabilities offered by the Scientific Machine Learning (SciML) ecosystem. Julia provides a suite of packages for symbolic-numeric computations, facilitating tasks like automatic sparsification and parallelisation, which enhance model performance and efficiency, and the tool aims to make these features accessible to the systems biology community.
– MakeSBML [11] is a web-based tool designed to facilitate the creation, editing, and searching of SBML-based models within the Biomodels repository. It enables users to convert models expressed in the human-readable Antimony language into SBML, and vice versa.
– MetaLo [12] is an open-source Python package designed to facilitate the integration of Boolean models, inferred from process description MIMs, with standard metabolic networks. It takes cell- and/or disease-specific molecular interaction maps in the CellDesigner XML file format and a generic constraint-based metabolic network in SBML. MetaLo helps to investigate signaling cascades, gene regulation mechanisms, and the distribution of metabolic fluxes in primary energy production pathways, and can manage both large-scale Boolean models and genome-scale metabolic models.
